# Comparative Study on Feature Selection in Protein Structure and Function Prediction

**DOI:** 10.1155/2022/1650693

**Published:** 2022-10-11

**Authors:** Wenjing Yi, Ao Sun, Manman Liu, Xiaoqing Liu, Wei Zhang, Qi Dai

**Affiliations:** ^1^College of Life Sciences, Zhejiang Sci-Tech University, Hangzhou 310018, China; ^2^College of Informatics Science and Technology, Zhejiang Sci-Tech University, Hangzhou 310018, China; ^3^College of Sciences, Hangzhou Dianzi University, Hangzhou 310018, China

## Abstract

Many effective methods extract and fuse different protein features to study the relationship between protein sequence, structure, and function, but different methods have preferences in solving the research of protein structure and function, which requires selecting valuable and contributing features to design more effective prediction methods. This work mainly focused on the feature selection methods in the study of protein structure and function, and systematically compared and analyzed the efficiency of different feature selection methods in the prediction of protein structures, protein disorders, protein molecular chaperones, and protein solubility. The results show that the feature selection method based on nonlinear SVM performs best in protein structure prediction, protein solubility prediction, protein molecular chaperone prediction, and protein solubility prediction. After selection, the accuracy of features is improved by 13.16% ~71%, especially the Kmer features and PSSM features of proteins.

## 1. Introduction

Protein structure and function is the basic research field of protein research, which is of great significance for the study of protein folding rate, DNA binding sites, and protein folding recognition [1–7]. In recent years, the gap between protein sequence and protein structure is becoming larger and larger with the development of sequencing technology, and the speed of identifying protein structure and function through experimental methods is relatively slow. Therefore, it is necessary to develop computational methods to quickly and accurately determine protein structure and function.

The function of a protein is determined by its spatial structure, which is determined by its sequence. Therefore, sequence information can be used to predict protein structure and function directly, so as to further guide biological experiments and reduce experimental costs. After the concept of protein structure class was put forward, several protein structure and function prediction methods were proposed [3–5, 7–11]. Some methods use protein composition information to predict protein structure and function [1, 12, 13]. For example, short peptide composition [14–16], pseudo amino acid composition [17–20], and functional domain composition match [21]. The sequence characteristic information is expressed as amino acid composition (AAC) by calculating the ratio of 20 amino acid residues in the sequence [14–16], but it does not take into account the physicochemical properties and interaction of amino acids. In order to overcome the above problems, pseudo amino acid composition (PseACC) calculates the composition of amino acid residues based on the hydrophobicity and other physical and chemical properties of amino acid residues [17–23].

The above methods are outstanding in high similarity data, but for low similarity data, their performance is ordinary, with prediction accuracy 50%. Therefore, we need to design more effective prediction algorithm. Kurgan et al. predicted protein secondary structures and designed SCPRED method on this basis [24]. Zhang et al. calculated the TPM matrix and took it as the characteristic representation of the protein secondary structures [25]. Dai et al. statistically analyzed the characteristic distribution of protein secondary structures and applied them to protein structure prediction [26]. Ding et al. constructed a multidimensional representation vector of protein secondary structure features, and fused it with existing features to achieve protein structure prediction [27]. Chen et al. and Kumar et al. combined structural information with physical and chemical characteristics to design a protein structure prediction method [28, 29]. Nanni et al. calculated the primary sequence characteristics and secondary structure characteristics of protein, respectively, for protein structure and function prediction [30]. Wang et al. simplified PSSM features and combined them with protein secondary structure features for protein structure prediction [31].

Through the fusion of the above features, the prediction accuracy of some methods on low similarity data sets has been improved to more than 80%, but there are still some problems in the development of protein structure and function prediction. In order to improve the prediction accuracy and efficiency of the model, the existing research is mainly achieved by fusing different types of protein features. However, it is worth noting that a simple combination of different features does not necessarily improve the prediction performance. If the combination is not appropriate, it may even offset the information contained in each other, which will not only lead to information redundancy but also increase the complexity and calculation of the model. This requires selecting valuable and contributing features, and then effective fusion, in order to design more effective prediction methods of protein structure and function.

With the above problems in mind, we introduced 16 feature selection methods based on mutual information, feature selection based on support vector machine, feature selection based on genetic algorithm, feature selection based on kurtosis and skewness, ReliefF, and sequentialfs information selection, and systematically compared their performance in protein structure class prediction, protein disorder prediction, protein molecular chaperone prediction, and protein solubility prediction. Through a comprehensive comparison and discussion, some novel valuable guidelines for use of the feature selection method for protein structure and function prediction are obtained.

## 2. Materials and Methods

### 2.1. Datasets

Four standard data sets for protein structure and function prediction were used in this work, which are protein structural class data set, molecular chaperone data set [32], solubility data set [33], and protein disorder data set [34]. The structure data set consists of 278 *α* structural proteins and 192 *β* proteins composition of structure. The molecular chaperone data set [35] is composed of 109 proteins that need Dnak/GroEL molecular chaperones to fold correctly, and 39 proteins that can fold autonomously. The solubility data set [36] is composed of 1000 proteins with high solubility and 1000 proteins with low solubility. The protein disorder data set is composed of 630 disordered proteins from DisProt and 3347 structural proteins from SCOP [37]. The detailed information of the data set is shown in Table 1.

### 2.2. Sequence Feature

Six kinds of different characteristic information of proteins are extracted [26]. They are Kmer, Pseudo Amino Acid Composition (PseAAC), Correlation-based features (correlation), composition-transition-distribution (RCTD), order-based features (order), position-based features (position), GO, and position-specific score matrix (PSSM).

### 2.3. Feature Selection

#### 2.3.1. Feature Selection Based on Mutual Information

Feature selection based on mutual information has become more and more popular in data mining, especially because of their ease of use, effectiveness, and strong theoretical foundation rooted in information theory. We adopted nine feature selection algorithms based on mutual information [38], which are maxRelFS, MRMRFS, minRedFS, MIQFS, QPFSFS, SPECCMI_Fs, MRMTRFS, CMIMFSand, and CIFEFS. The common point of these methods is that they all focus on the concepts of redundancy and correlation, and use greedy schemes to build the selected feature sets incrementally. Given a sample, the column is the characteristic matrix *X*, and the corresponding category is *C*. The calculation formula of mutual information is

(1) $$\begin{array}{c} Rel\left(X_{i}\right)=I\left(X_{i};C\right)=\underset{X_{i},C}{\sum }P\left(X_{i},C\right)\log\frac{P\left(X_{i},C\right)}{P\left(X_{i}\right)P\left(C\right)}. \end{array}$$

If the selected set is *S*, the calculation formula of redundancy is as follows:

(2) $$\begin{array}{c} Red\left(X_{i}\left|S\right.\right)=\frac{1}{S}\underset{X_{j}\in S}{\sum }I\left(X_{i};X_{j}\right). \end{array}$$

The above nine feature selection algorithms calculate the mutual information value of each feature and category *C*, and select the feature with the largest mutual information as the optimal feature. Then, according to the feature selection method of quadratic programming, the features with minimum redundancy and maximum correlation are selected one by one. Finally, we can get a feature vector sorted according to the importance of features.

#### 2.3.2. Support Vector Machine Recursive Feature Extraction (SVM-RFE)

Support vector machine recursive feature extraction (SVM-RFE) is divided into linear SVM-RFE and nonlinear SVM-RFE. The details are as follows:

*(1) Linear SVM-RFE*. For a samples {x_i_, yi}, the objective function of linear SVM-RFE is

(3) $$\begin{array}{c} f\left(x\right)=a\cdot x+b, \end{array}$$

where *a* is weight factor and *b* is deviation. Thus, the Lagrangian version of this problem can be expressed as

(4) $$\begin{array}{c} L_{D}=\overset{n}{\underset{i=1}{\sum }}\alpha_{i}-\frac{1}{2}\overset{n}{\underset{i,j=1}{\sum }}\alpha_{i}\alpha_{j}y_{i}y_{j}x_{i}x_{j}, \end{array}$$

where *α*_i_ is Lagrange factor. *α*_i_ can be calculated by LD maximum under the condition of *α*_i_ ≥0 and ∑_*i*=1_^*n*^*α*_*i*_*y*_*i*_ = 0. Weighting factors can be calculated by the following formula:

(5) $$\begin{array}{c} a=\overset{n}{\underset{i=1}{\sum }}\alpha_{i}y_{i}x_{i}. \end{array}$$

*k*-th feature sorting criteria is the square of the *k*-th weighting factor.

(6) $$\begin{array}{c} J\left(k\right)=w_{k}^{2}. \end{array}$$

In the training process, the feature with the smallest influence factor will be deleted every time, and so on, until all the features are deleted. Then, the importance of features is sorted according to the order in which they are deleted [39].

*(2) Nonlinear SVM-RFE*. In many cases, the number of features of the sample will be more than the number of samples. At this time, using linear SVM-RFE can avoid the phenomenon of over fitting [40]. However, when the number of samples is greater than the number of features, the selection result of nonlinear SVM-RFE will be better than that of linear SVM-RFE.

Nonlinear SVM-RFE will map features to new spaces with higher dimensions as follows:

(7) $$\begin{array}{c} x\in R^{d}\mapsto \varphi \left(x\right)\in R^{h}. \end{array}$$

In the new space, the samples are expected to be linearly separable. Its Lagrangian form can be expressed as

(8) $$\begin{array}{c} L_{D}=\overset{n}{\underset{i=1}{\sum }}\alpha_{i}-\frac{1}{2}\overset{n}{\underset{i,j=1}{\sum }}\alpha_{i}\alpha_{j}y_{i}y_{j}\varphi \left(x_{i}\right)\varphi \left(x_{j}\right). \end{array}$$

Thus, we could transform inner product *φ*(*x*_*i*_)*φ*(*x*_*j*_) into a Gaussian kernel *K*(*x*_*i*_, *x*_*j*_) as follows:

(9) $$\begin{array}{c} K\left(x_{i},x_{j}\right)=\ell^{-\lambda {\left\|x_{i}-x_{j}\right\|}^{2}.} \end{array}$$

Thus, *k*-th feature sorting criteria could be expressed as

(10) $$\begin{array}{c} J\left(k\right)=\frac{1}{2}\overset{n}{\underset{i,j=1}{\sum }}\alpha_{i}\alpha_{j}y_{i}y_{j}K\left(x_{i},x_{j}\right)-\frac{1}{2}\overset{n}{\underset{i,j=1}{\sum }}\alpha_{i}\alpha_{j}y_{i}y_{j}K\left(x_{i}^{\left(-k\right)},x_{j}^{\left(-k\right)}\right). \end{array}$$

*x*_*i*_^(−*k*)^ represents that feature *k* has been removed.

#### 2.3.3. Feature Selection Based on Genetic Algorithm

We adopted the assembled neural network (ASNN) algorithm. This method carries out combinatorial optimization by using the idea of genetic algorithm. For a given data set, a behavior sample can be constructed and listed as the matrix *X* of features [41], and finally a feature vector will be obtained, which is the optimal feature set, but the ranking of each feature is not related to its importance.

#### 2.3.4. Feature Selection Based on Kurtosis and Skewness

For a vector of length n {x_1_, x_2_, ..., x_n_}, its kurtosis and skewness are calculated as follows:

(11) $$\begin{array}{c} \text{Kurtosis}=\frac{\sum_{i=1}^{n}{\left(x_{i}-\bar{x}\right)}^{4}}{\left(n-1\right)SD^{4}}-3,\\ \text{skewness}=\frac{\sum_{i=1}^{n}{\left(x_{i}-\bar{x}\right)}^{3}}{\left(n-1\right)SD^{3}}. \end{array}$$

Kurtosis and skewness are statistics used to measure the distribution of data. In this work, we calculated the skewness and kurtosis of each feature, and then sort them according to their values as a method to measure the importance of features.

#### 2.3.5. Relieff Algorithm

Relieff algorithm randomly takes a sample *R* from the training sample set every time, then finds *k* nearest neighbor samples of *R* from the sample set of the same kind as *R*, and finds *k* nearest neighbor samples from the sample set of different classes of each *R*, and then updates the weight of each feature. The formula is as follows:

(12) $$\begin{array}{c} W\left(A\right)=W\left(A\right)-\overset{k}{\underset{j=1}{\sum }}\frac{\text{diff}\left(A,R,H_{j}\right)}{mk}+\underset{C\notin \text{class}\left(R\right)}{\sum }\frac{p\left(C\right)/1-p\left(\text{Class}\left(R\right)\right)\sum_{j=1}^{k}diff\left(A,R,M_{j}\left(C\right)\right)}{mk}, \end{array}$$

where diff(*A*, *R*1, *R*2) means the difference between feature *R1* and *R2* in feature *A*. *M*_*j*_(*C*) means the *j*-th nearest neighbor sample in class *C*. Formula is as follows:

(13) $$\begin{array}{c} \text{diff}\left(A,R1,R2\right)=\left\{\begin{array}{cc} \frac{\left|R_{1}\left[A\right]-R_{2}\left[A\right]\right|}{\max\left(A\right)-\min\left(A\right)} & \text{if}\;A\;\text{is}\;\text{consequent},\\ 0 & \text{if}\;A\;\text{is}\;\text{unconsequent}\;\text{and}\;R_{1}\left[A\right]=R_{2}\left[A\right],\\ 1 & \text{if}\;A\;\text{is}\;\text{unconsequent}\;\text{and}\;R_{1}\left[A\right]\neq R_{2}\left[A\right]. \end{array}\right. \end{array}$$

#### 2.3.6. Sequentialfs

We adopted the forward feature selection algorithm of sequence in this work. For a training set {*x*_train_*,y*_train_} and validation set {*x*_validation_*,y*_validation_}, the evaluation criteria can be expressed as

(14) $$\begin{array}{c} {\left\|y_{\text{validation}}-x_{\text{validation}}\frac{x_{\text{train}}}{y_{\text{train}}}\right\|}_{2}. \end{array}$$

### 2.4. Classification Algorithm

Support vector machine is a large-scale edge classifier based on statistical learning theory [42]. It uses the optimal separation hyperplane to separate two kinds of data. For binary support vector machines, the decision function is

(15) $$\begin{array}{c} f\left(x\right)=\overset{N}{\underset{i=1}{\sum }}\alpha_{i}y_{i}K\left(x_{i},x\right)+b. \end{array}$$

where *b* is a constant, *C* is a cost parameter controlling the trade-off between allowing training errors and forcing rigid margins, y_*i*_*ϵ*{−1, +1}, *x*_*i*_ is the support vector, 0 ≤ *α*_*i*_ ≤ *C*, and *K*(*x*_*i*_, *x*) is the kernel function. This work chooses the Gauss kernel function of support vector machine because of its superiority in solving nonlinear problems [34, 37]. Furthermore, a simple grid search strategy is used to select the parameters *C* and gamma with the highest overall prediction. It is designed based on 10 times cross validation of each dataset, and the values of *C* and gamma are taken from the 2^−10^ to 2^10^.

### 2.5. Performance Evaluation

This work adopted different feature selection methods for different data sets, and used the leave one method for evaluation. Finally, the prediction results are compared by calculating accuracy.

For each data set, we compared the efficiency of different feature selection methods through the following steps. The following takes the feature selection method based on genetic algorithm (GA) and PSSM features as examples to introduce the evaluation process:

1. PSSM information is selected by GA feature selection method
2. Select the top 10, 20, 30, 40, and 50 features using GA (if the number of features is insufficient, all the information will be taken out), input them into SVM classifier for classification prediction, and calculate the accuracy of prediction ACC1, ACC2, ACC3, ACC4, and ACC5
3. Subtract the accuracy of the whole PSSM information from ACC1, ACC2, ACC 3, ACC 4, and ACC 5
4. Compare the changes in accuracy of various special products after 16 selection methods

We also compared and analyzed the characteristics of selection, and the main steps are as follows:

1. Use the above 16 selection methods to select each type of feature
2. According to the selection results of 16 feature selection methods, the importance of each type of feature is ranked
3. Take out the first 10, 20, 30, 40, and 50 features of each type of feature, respectively, (if the number of features is insufficient, all the information will be taken out) and mix them together as five new mixed features (*I*_10_, *I*_20_, *I*_30_, *I*_40_, *I*_50_);
4. Then, 16 feature selection methods are used to select the mixed features
5. According to the results of the fourth step, the importance of the fused features is ranked
6. Take out the top 10 features and count the type of features from which these 10 features come. Take out the top 20 features and count the categories of features
7. In five cases, if there are a large number of certain features (or observed), it means that such features are more important

## 3. Results and Discussion

### 3.1. Comparison of Feature Selection in Protein Structure Prediction

**Figure 1:**
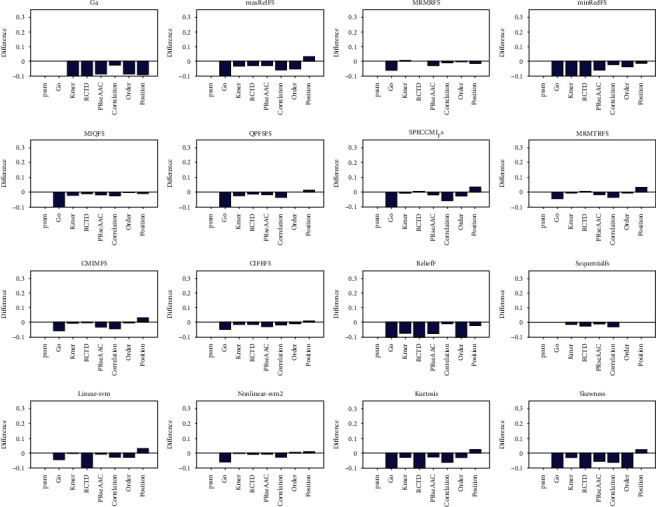
The comparison between the accuracy of support vector machine prediction and that of single class feature prediction after selecting the top 10 features. For each graph, the selection method is arranged from left to right and from top to bottom. They are GA, and there are nine selection methods of mutual information, relief, sequentialfs, linear SVM, nonlinear SVM, kurtosis, and skewness. The horizontal axis represents sequence features, which are PSSM, go, Kmer, RCTD, PRseAAC, correlation, order, and position, respectively.

We first discussed the efficiency of different feature selection methods in protein structure prediction. We adopted the structural data set, which contains 278 items *α* structural proteins and 192 *β* structural proteins. In this work, eight kinds of features are selected through 16 feature selection methods, and the selected features are input into the support vector machine to predict the structural class of protein. The quality of feature selection methods is evaluated based on the accuracy of prediction, which are represented in Figure 1 and Supplementary Figures 1–4.

From Figure 1 and Supplementary Figures 1–4, it is easy to note that the accuracy of MRMRFS, MRMTRFS, CMIMFS, CIFEFS, and nonlinear SVM feature selection methods changes the most, and the change range is 3.19% for the position feature. By comparing the accuracy of the first 20-50 features selected with that of the unselected features, it can be seen that the biggest change in accuracy is the GO features selected by nonlinear SVM, with changes of 2.13%, 6.39%, 6.17%, and 4.68%, respectively. Therefore, nonlinear SVM feature selection method performs best in protein structure prediction.

**Figure 2:**
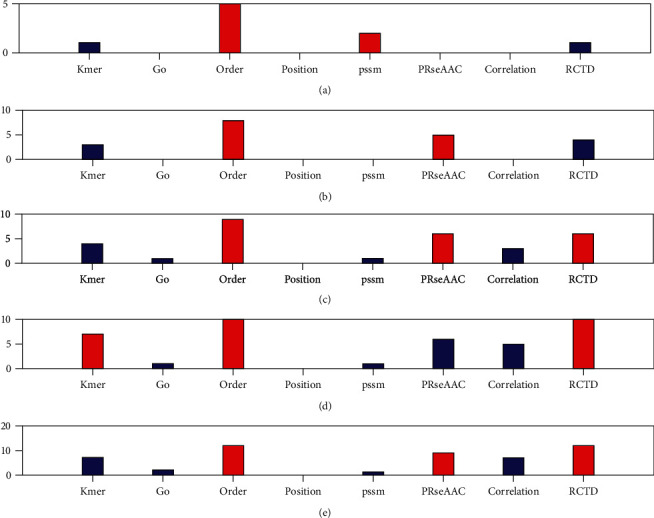
The number of 8 types of features in the top selected features in the protein structural data. From (a) to (e), it means that the number of selected features is 10 to 50, respectively.

For structural data sets [43], we further compared and analyzed the types of selected features. First, eight types of features are fused, and the fused features are selected through 16 feature selection methods, and the top 10-50 features are selected. Then, the number of eight types of features in the top 10-50 total selected features is counted, and the preference of eight types of features is evaluated by proportion.  Figure 2 shows the number of 8 types of features in the top 10-50 total selected features in the protein structural data.

Figure 2 show that when the total number of selections is 10, there are 5 order features, accounting for 50%. When the total selection number is 20, there are 8 order features, accounting for 40%. When the selection number is 40, both order and RCTD have 10, accounting for 25% of the top 40 features. When the total selection number is 50, there are 12 orders and RCTD features, respectively, accounting for 24% of the total. The above results show that order feature is the first choice for protein structure prediction, followed by RCTD feature.

### 3.2. Comparison of Feature Selection in Protein Disorder Prediction

**Figure 3:**
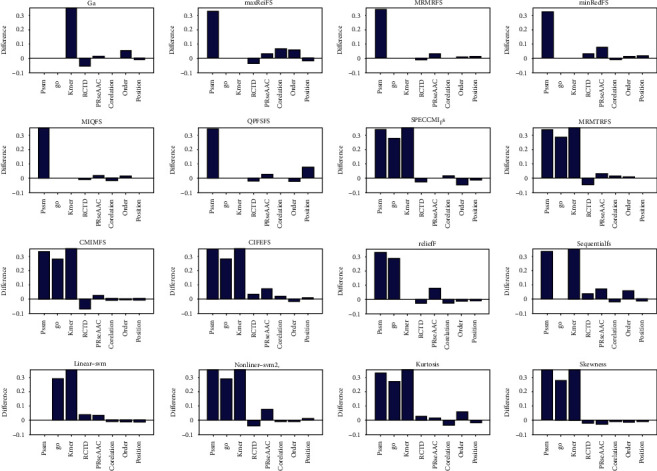
The comparison between the accuracy of support vector machine prediction and that of single class feature prediction after selecting the top 10 features. For each graph, the selection method is arranged from left to right and from top to bottom. They are GA, and there are nine selection methods of mutual information, relief, sequentialfs, linear SVM, nonlinear SVM, kurtosis and skewness. The horizontal axis represents sequence features, which are PSSM, go, Kmer, RCTD, PRseAAC, correlation, order, and position, respectively.

We then discussed the efficiency of different feature selection methods in protein disorder prediction. The protein disorder data set [44] used in this chapter is from two protein databases related to structural classes, including 630 disordered proteins from disProt and 3347 structural proteins from SCOP. In this work, eight kinds of features are selected through 16 feature selection methods, and the selected features are input into KNN to predict protein disorder. The quality of feature selection methods is evaluated based on the accuracy of prediction, which are represented in Figure 3 and Supplementary Figures 5–8.

It can be seen from Figure 3 and Supplementary Figures 5–8 that when PSSM feature, go feature and Kmer feature are input into KNN algorithm for prediction, the change values of their accuracy are 51.28%, 55.11% and 26.95%, respectively. It can be seen that after feature selection, the accuracy of protein disorder prediction is significantly improved. When selecting 10 features, SPECCMI_FS performs best based on Kmer feature, and its accuracy by 71%. When selecting the first 20 and 30 features, the nonlinear SVM feature selection method is particularly prominent in Kmer features, and its accuracy has increased by 64.19%. Among the top 40 features selected, CIFEFS selection method performs best in Kmer features, and the accuracy is improved to 65.21%. Among the top 50 features selected, CIFEFS and linear SVM selection methods are outstanding, and the accuracy has increased by 59.61%. The above results show that for protein disorder data sets, SPECCMI_FS, CIFEFS, nonlinear SVM, and linear SVM feature selection methods can select core features from Kmer features, which improve its accuracy by 59.61% ~71%.

**Figure 4:**
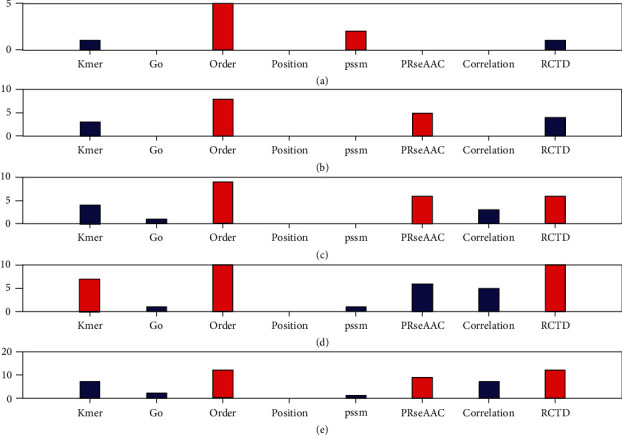
The number of 8 types of features in the top selected features in the protein structural data. From (a) to (e), it means that the number of selected features is 10 to 50, respectively.

We also further compared the types of selected features. First, eight types of features are fused, and the fused features are selected through 16 feature selection methods, and the top 10-50 features are selected. Then, the number of eight types of features in the top 10-50 total selected features is counted, and the preference of eight types of features is evaluated by proportion.  Figure 4 shows the number of 8 types of features in the top 10-50 total selected features in the protein disorder data set.

Figure 4 shows the number of features selected at five levels from top to bottom. If the top 10 fusion features are selected, 5 of them are from order features. If the first 20 fusion features are selected, 8 of them are from order features. If the first 30 fusion features are selected, 9 of them are from order features. If the first 40 fusion features are selected, there are 10 features from order and RCTD, respectively. If the top 50 fusion features are selected, 12 of them are from order and RCTD features, respectively. Therefore, the order and RCTD feature will help to improve the accuracy of the protein disorder prediction.

### 3.3. Comparison of Feature Selection in Protein Molecular Chaperone Prediction

**Figure 5:**
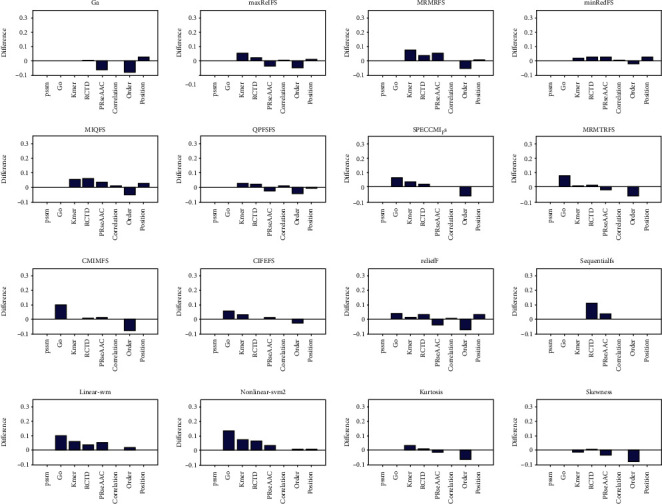
The comparison between the accuracy of support vector machine prediction and that of single class feature prediction after selecting the top 10 features. For each graph, the selection method is arranged from left to right and from top to bottom. They are GA, and there are nine selection methods of mutual information, relief, sequentialfs, linear SVM, nonlinear SVM, kurtosis, and skewness. The horizontal axis represents sequence features, which are PSSM, go, Kmer, RCTD, PRseAAC, correlation, order, and position, respectively.

We then discussed the efficiency of different feature selection methods in protein molecular chaperone prediction. In the data set used in this work, there are 109 proteins that need Dnak/GroEL molecular chaperones to fold correctly, and the remaining 39 proteins that can fold autonomously. In this work, eight kinds of features are selected through 16 feature selection methods, and the selected features are input into KNN to predict protein disorder. The quality of feature selection methods is evaluated based on the accuracy of prediction, which are represented in Figure 5 and Supplementary Figures 9–12.

Figure 5 and Supplementary Figures 9–12 show that when selecting the top 10 and 20 features, the accuracy of GO feature selection using nonlinear SVM is improved by 13.16% and 14.48%. When selecting the first 30 and 50 features, the accuracy of using sequentialfs to select RCTD features is improved by 13.16% and 17.17%. When selecting the first 40 features, linear SVM is used to select Kmer features, which improves its accuracy by 14.48%. Therefore, nonlinear SVM, sequentialfs and linear SVM are used to select features in the molecular chaperone prediction, which improves its accuracy by 13.16% ~17.17%.

**Figure 6:**
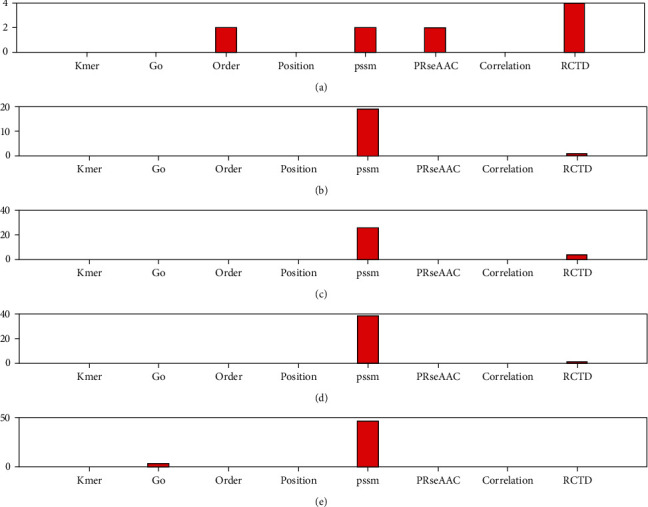
The number of 8 types of features in the top selected features in the protein structural data. From (a) to (e), it means that the number of selected features is 10 to 50, respectively.

We also further compared the types of selected features. First, eight types of features are fused, and the fused features are selected through 16 feature selection methods, and the top 10-50 features are selected. Then, the number of eight types of features in the top 10-50 total selected features is counted, and the preference of eight types of features is evaluated by proportion.  Figure 6 shows the number of 8 types of features in the top 10-50 total selected features in the protein disorder data set.

When selecting 10 comprehensive features, there are 5 RCTD features, accounting for 50%. When selecting 20-30 comprehensive features, PSSM features have an absolute advantage, with 19, 26, 39, and 47 selected, respectively. It can be seen that PSSM is the preferred feature if you want to check whether a protein sequence is self-folding or molecular chaperone to help complete the correct folding.

### 3.4. Comparison of Feature Selection in Protein Solubility Prediction

**Figure 7:**
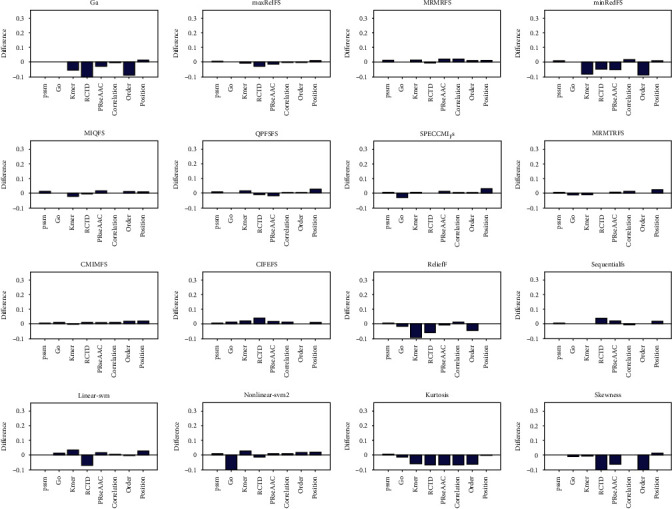
The comparison between the accuracy of support vector machine prediction and that of single class feature prediction after selecting the top 10 features. For each graph, the selection method is arranged from left to right and from top to bottom. They are GA, and there are nine selection methods of mutual information, relief, sequentialfs, linear SVM, nonlinear SVM, kurtosis, and skewness. The horizontal axis represents sequence features, which are PSSM, go, Kmer, RCTD, PRseAAC, correlation, order, and position, respectively.

Finally, the efficiency of different feature selection methods in protein solubility prediction is discussed. In this work, more than 7000 proteins from E. coli were selected and sorted according to their solubility. The first 1000 protein sequences with higher solubility and the last 1000 protein sequences with the lowest solubility were taken out to form a protein sequence data set. Through 16 feature selection methods, 8 kinds of features are selected, respectively, and the selected features are input into KNN to predict the solubility of protein. The quality of feature selection methods is evaluated based on the accuracy of prediction, which are represented in Figure 7 and Supplementary Figures 13–16.

When selecting 10 and 20 features, using CIFEFS based on mutual information to select RCTD features, the accuracy is improved the most, which is 3.93% and 3.88%, respectively. When selecting 30 features, using sequentialfs to select RCTD features, the accuracy is improved by 3.12%. When 40 and 50 features are selected, the accuracy of nonlinear SVM is improved by 3.12% and 4.76%, respectively. The above results show that CIFEFS, sequentialfs and nonlinear SVM feature selection methods perform well in protein solubility prediction.

**Figure 8:**
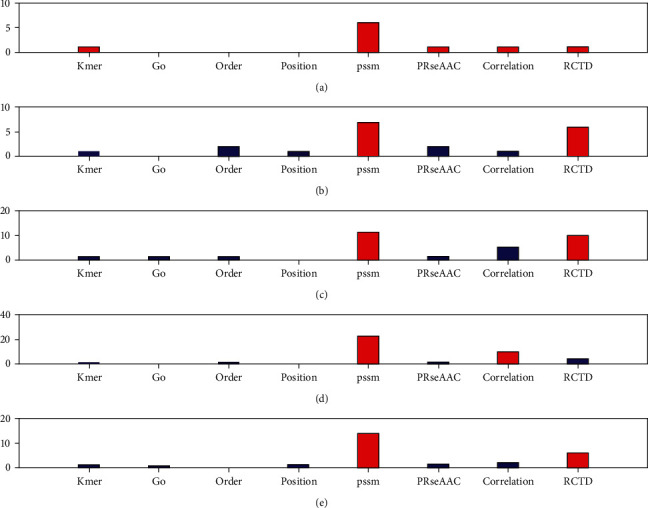
The number of 8 types of features in the top selected features in the protein structural data. From (a) to (e), it means that the number of selected features is 10 to 50, respectively.

We also further compared the types of selected features. First, eight types of features are fused, and the fused features are selected through 16 feature selection methods, and the top 10-50 features are selected. Then, the number of eight types of features in the top 10-50 total selected features is counted, and the preference of eight types of features is evaluated by proportion.  Figure 8 shows the number of 8 types of features in the top 10-50 total selected features in the protein disorder data set.

When selecting 10-50 comprehensive features, PSSM features always account for the most, with 6, 7, 11, 23 and 28 PSSM features, accounting for 60%, 35%, 36.67%, 50.75% and 56% of the total. Therefore, using PSSM characteristics as input features to predict the solubility of new protein sequences is more reliable [45].

### 3.5. Comparison of Calculation Efficiency of Various Methods

The above analysis shows that the nonlinear SVM feature selection method based on support vector machine performs well in the prediction of various protein structures and functions. In order to further study the computational efficiency of feature selection methods, we calculated the time-consuming of various feature selection methods to select 8 types of features, as shown in Table 2. Mutual information represents the average time of the nine selection methods. It is not difficult to find that the nonlinear SVM selection method is related to the size of matrix elements. The larger the data elements, the longer the time required. Therefore, the matrix is normalized before feature selection. Sequentialfs consumes the most time, and the time-consuming ratio of nonlinear SVM, linear SVM, and single mutual information selection method is 2.5: 27.5 : 1. Therefore, the nonlinear SVM selection method is the preferred feature selection method in the prediction of protein structure and function.

## 4. Conclusion

Feature selection can reduce the problem of over fitting, improves the performance of the model, and reduces the time and space cost of the learning algorithm. 16 feature selection methods used in this work are feature selection method based on mutual information, feature selection method based on support vector machine, feature selection method based on genetic algorithm, feature selection method based on kurtosis and skewness, ReliefF ,and sequentialfs information selection methods. Different feature selection methods were compared and analyzed in protein structure class prediction, protein disorder prediction, protein molecular chaperone prediction, and protein solubility prediction.

Through a comprehensive comparison and discussion, we found that nonlinear SVM feature selection method performs best in protein structure prediction, the first choice is order feature, followed by RCTD feature. In protein disorder prediction, SPECCMI_FS, CIFEFS, nonlinear SVM, and linear SVM feature selection methods can select core features from Kmer features, which improves its accuracy by 59.61% ~71%. At the same time, order or RCTD features as input information will help to improve the accuracy of prediction. In protein molecular chaperone prediction, nonlinear SVM, sequentialfs, and linear SVM are used to select features, which improves the accuracy by 13.16% ~17.17%, and the preferred feature is PSSM feature. In protein solubility prediction, CIFEFS, sequentialfs, and nonlinear SVM feature selection methods perform well, and PSSM is the preferred feature. These results can be regarded as some novel valuable guidelines for use of the feature selection method for protein structure and function prediction.

## Figures and Tables

**Table 1:** Detailed information of protein structure and function data.

Dataset	Positive	Negative	Total
Protein structural class	278	192	470
Molecular chaperone	109	39	148
Solubility	1000	1000	2000
Protein disorder	630	3347	3977

**Table 2:** Time consumption of feature selection methods.

	Mutual Information (/S)	Sequentialfs (/S)	Linear-svm (/S)	Nonlinear-svm (/S)
PSSM	5.8	14074	23.8	2082.4
Go	360.33	—	42.4	0.75
RCTD	4.2	5571	11.7	1.3
Kmer	6.3	7423	18.9	1.7
PRseAAC	0.67	5.83	4.32	0.36
Order	1	35.2	22.8	270.5
Position	0.75	2.09	2.04	0.17
Correlation	0.62	3.87	1.39	0.33

## Data Availability

The data are available in https://github.com/bioinfo0706/RaaMLab.

## Supplementary Materials

Supplementary Materials Supplementary Figures 1‑16 are the precision comparison between support vector machine prediction and single class feature prediction based on the selected 20, 30, 40 and 50 features.
