# Supplementary material for: Comparative Study on Feature Selection in Protein Structure and Function Prediction
Source: Comput Math Methods Med. 2022 Oct 11;2022:1650693. doi: 10.1155/2022/1650693 (PMC9578875; doi:10.1155/2022/1650693)
Supplement: Supplementary Materials — Supplementary Figures 1‑16 are the precision comparison between support vector machine prediction and single class feature prediction based on the selected 20, 30, 40 and 50 features. [file 1650693.f1.docx]

## Comparative study on feature selection in protein structure and function prediction

**Wenjing Yi^1^, Ao Sun^2^, Manman Liu^2^, Xiaoqing Liu^3^, Wei Zhang^2*^, Qi Dai ^1^ ^[[1]](#footnote-0)^***

^1^ *College of Life Sciences, Zhejiang Sci-Tech University, Hangzhou 310018, People’s Republic of China*

*1 College of Informatics Science and Technology, Zhejiang Sci-Tech University, Hangzhou 310018, People’s Republic of China*

*3 College of Sciences, Hangzhou Dianzi University , Hangzhou 310018, People’s Republic of China*

1. **Supp Figure 1-Supp Figure 16.**


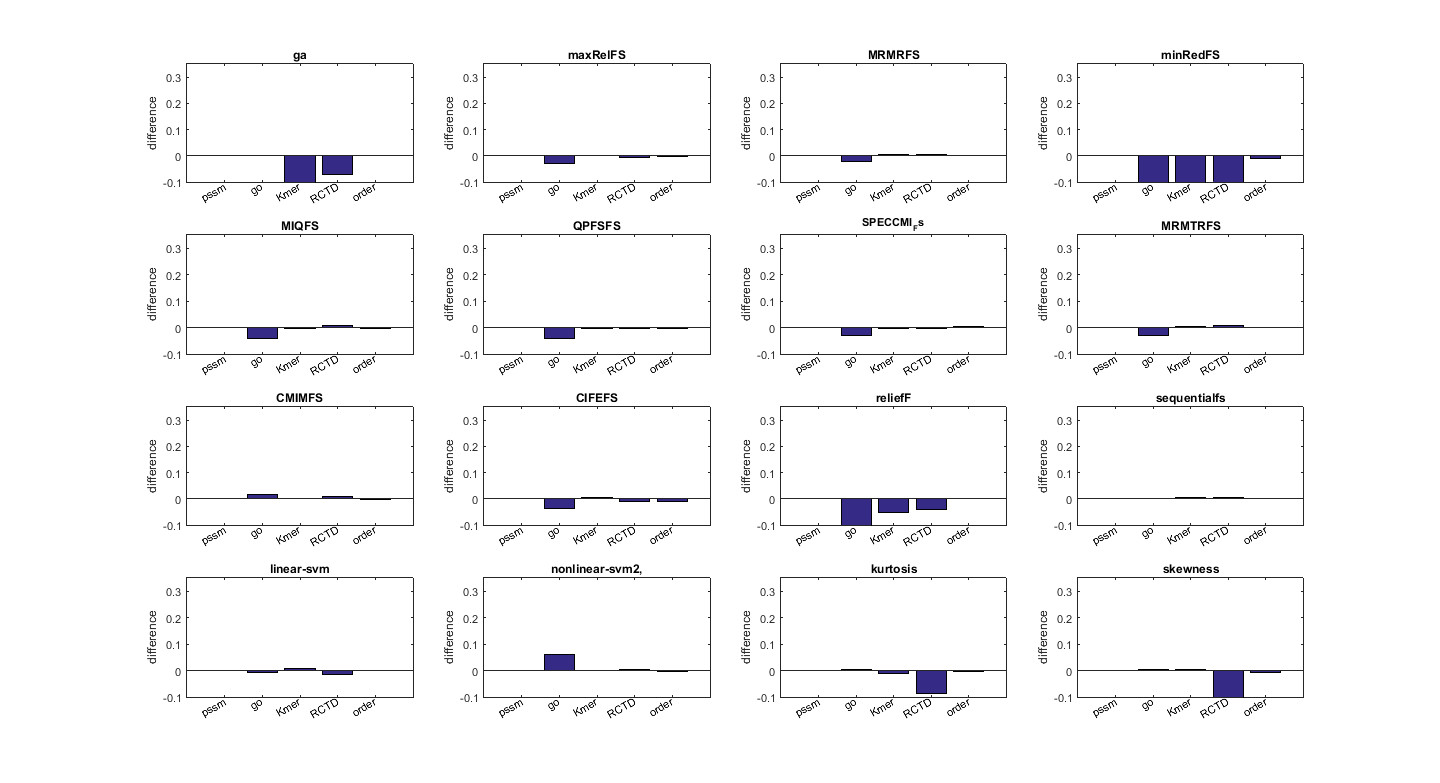


Supp Figure 1. The comparison between the accuracy of support vector machine prediction and that of single class feature prediction after selecting the top 20 features. For each graph, the selection method is arranged from left to right and from top to bottom. They are GA, and there are nine selection methods of mutual information, relief, sequentialfs, linear SVM, nonlinear SVM, kurtosis and sketchness. The horizontal axis represents sequence features, which are PSSM, go, Kmer, RCTD, PRseAAC, correlation, order and position respectively.


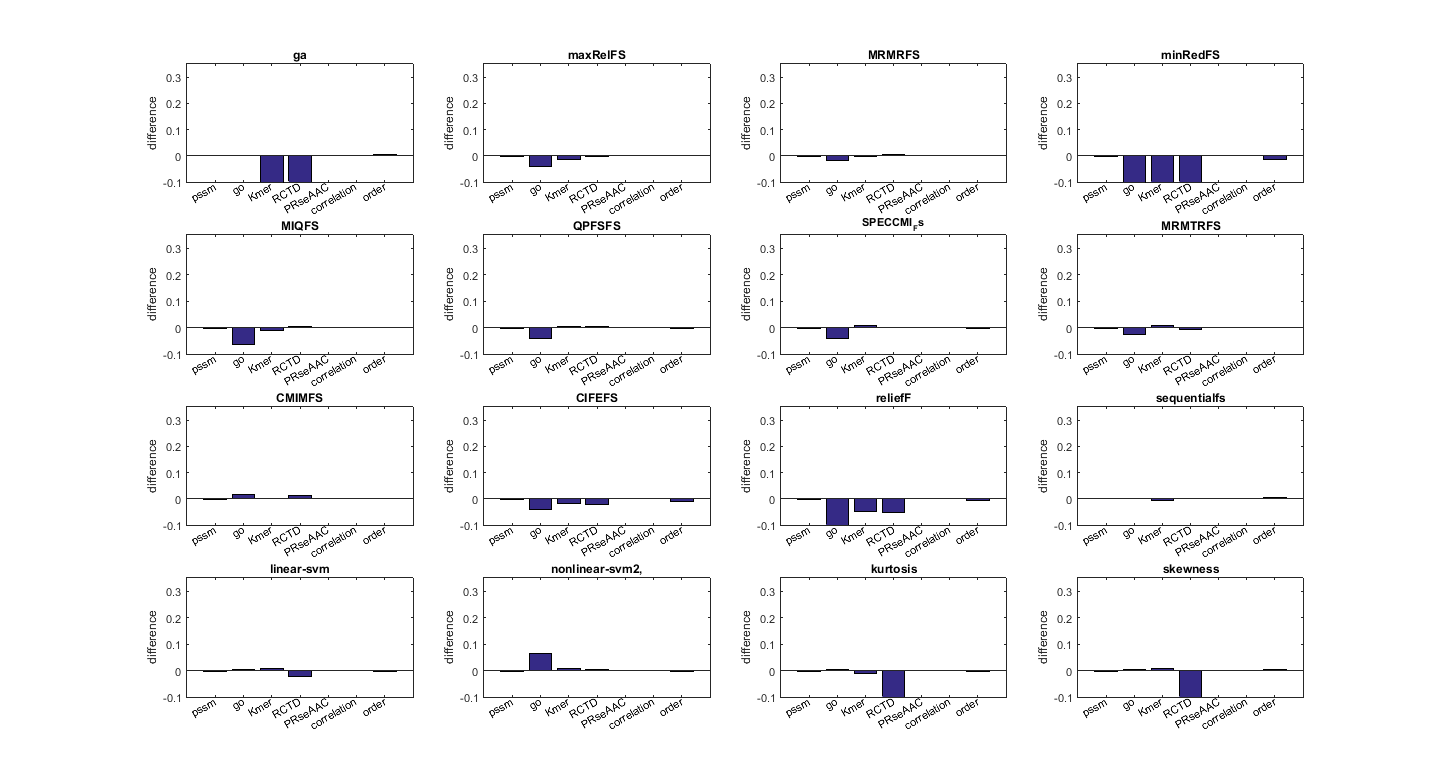


Supp Figure 2. The comparison between the accuracy of support vector machine prediction and that of single class feature prediction after selecting the top 30 features. For each graph, the selection method is arranged from left to right and from top to bottom. They are GA, and there are nine selection methods of mutual information, relief, sequentialfs, linear SVM, nonlinear SVM, kurtosis and sketchness. The horizontal axis represents sequence features, which are PSSM, go, Kmer, RCTD, PRseAAC, correlation, order and position respectively.


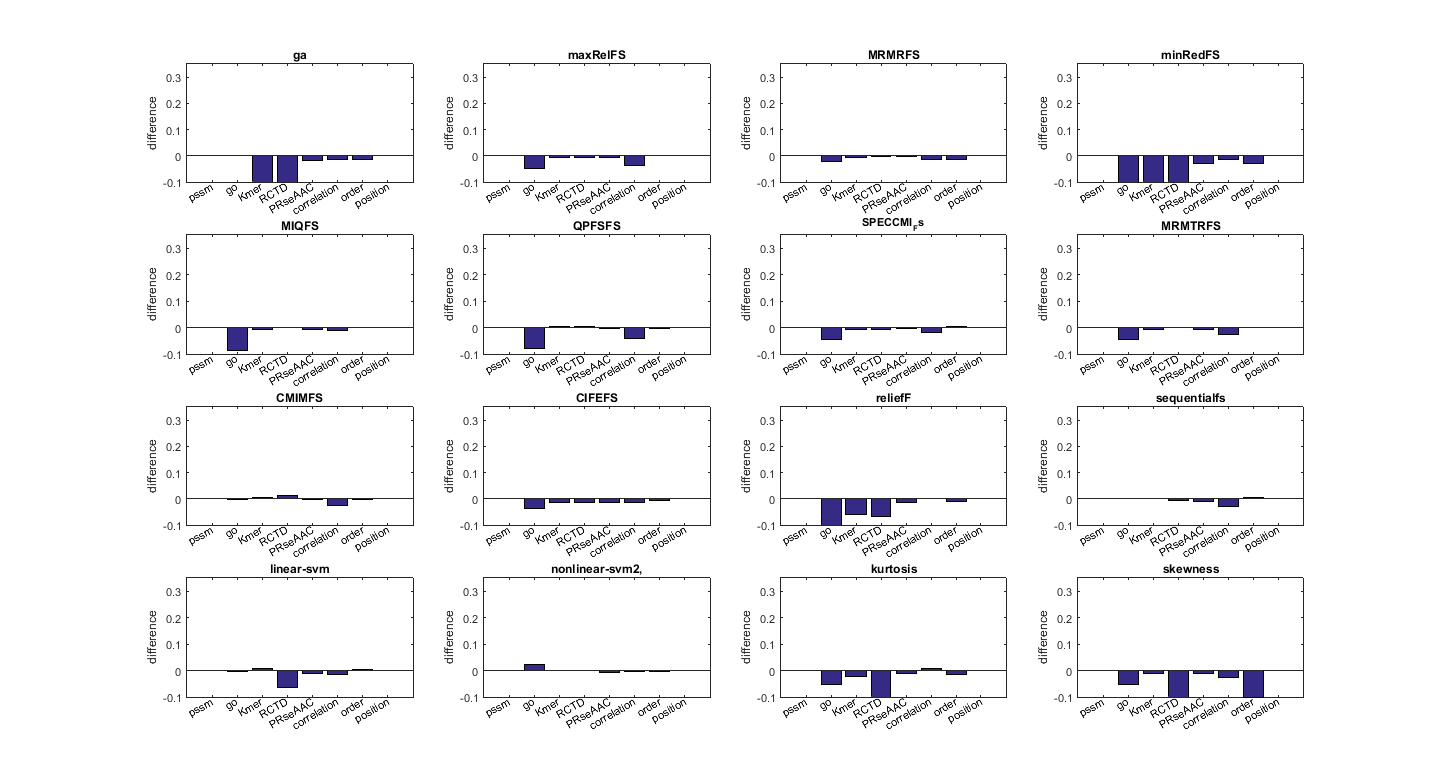


Supp Figure 3. The comparison between the accuracy of support vector machine prediction and that of single class feature prediction after selecting the top 40 features. For each graph, the selection method is arranged from left to right and from top to bottom. They are GA, and there are nine selection methods of mutual information, relief, sequentialfs, linear SVM, nonlinear SVM, kurtosis and sketchness. The horizontal axis represents sequence features, which are PSSM, go, Kmer, RCTD, PRseAAC, correlation, order and position respectively.


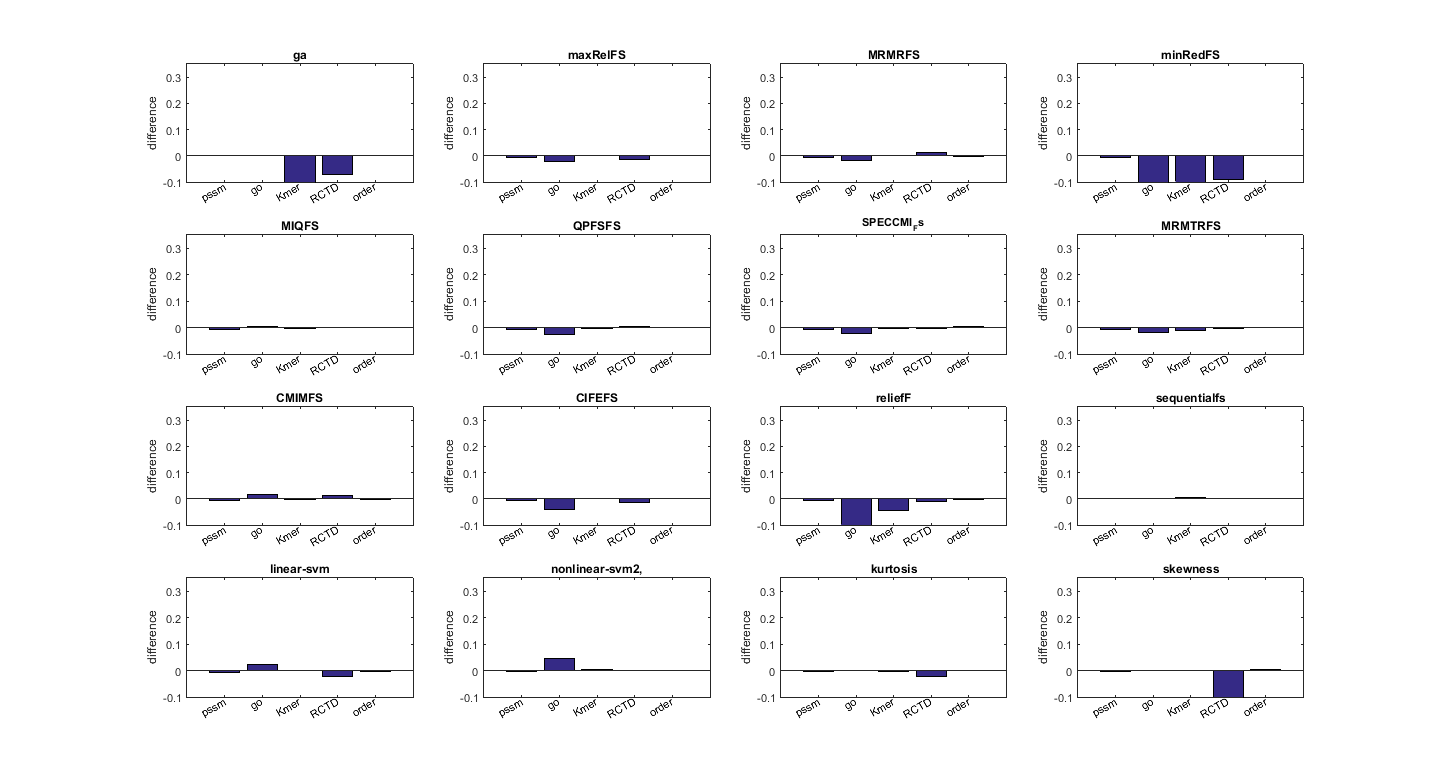


Supp Figure 4. The comparison between the accuracy of support vector machine prediction and that of single class feature prediction after selecting the top 50 features. For each graph, the selection method is arranged from left to right and from top to bottom. They are GA, and there are nine selection methods of mutual information, relief, sequentialfs, linear SVM, nonlinear SVM, kurtosis and sketchness. The horizontal axis represents sequence features, which are PSSM, go, Kmer, RCTD, PRseAAC, correlation, order and position respectively.


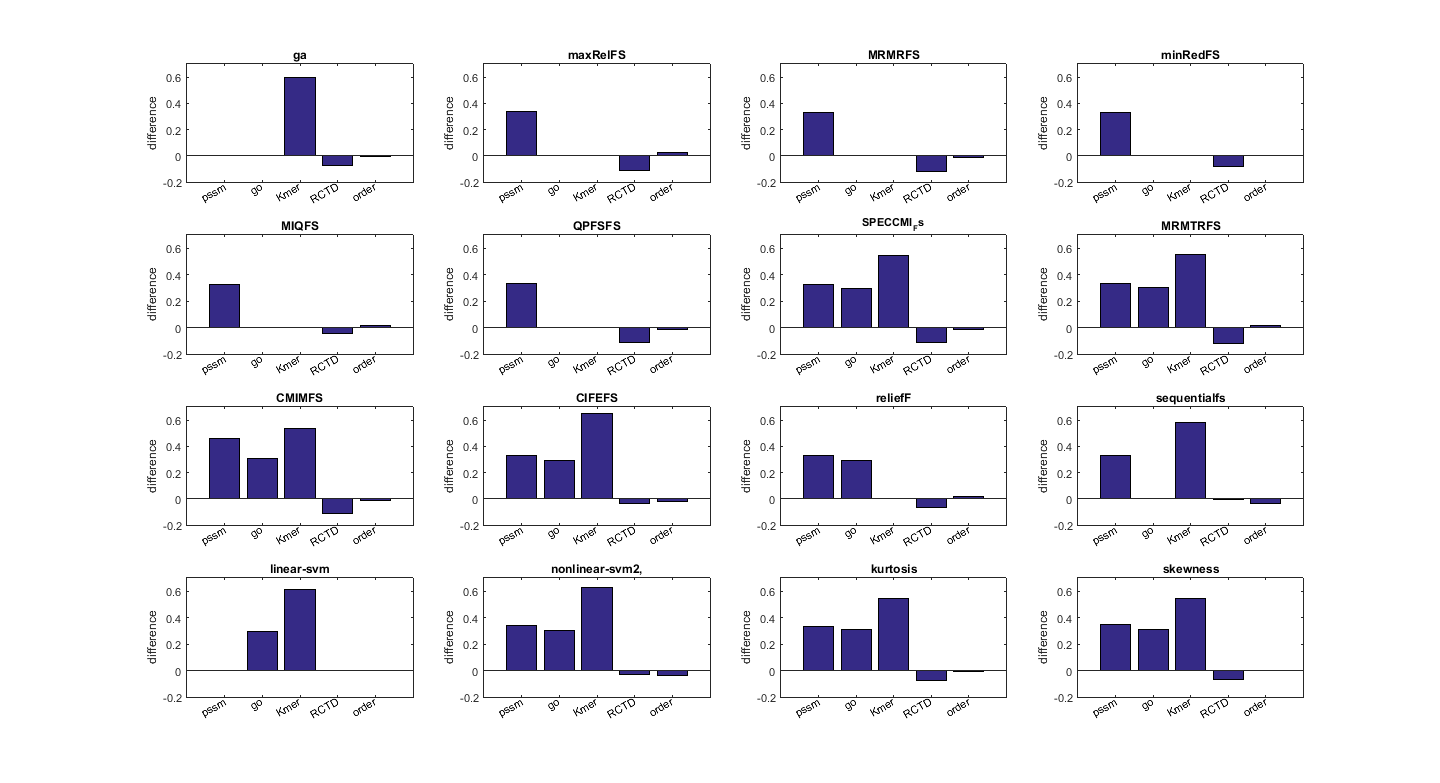


Supp Figure 5. The comparison between the accuracy of support vector machine prediction and that of single class feature prediction after selecting the top 20 features. For each graph, the selection method is arranged from left to right and from top to bottom. They are GA, and there are nine selection methods of mutual information, relief, sequentialfs, linear SVM, nonlinear SVM, kurtosis and sketchness. The horizontal axis represents sequence features, which are PSSM, go, Kmer, RCTD, PRseAAC, correlation, order and position respectively.


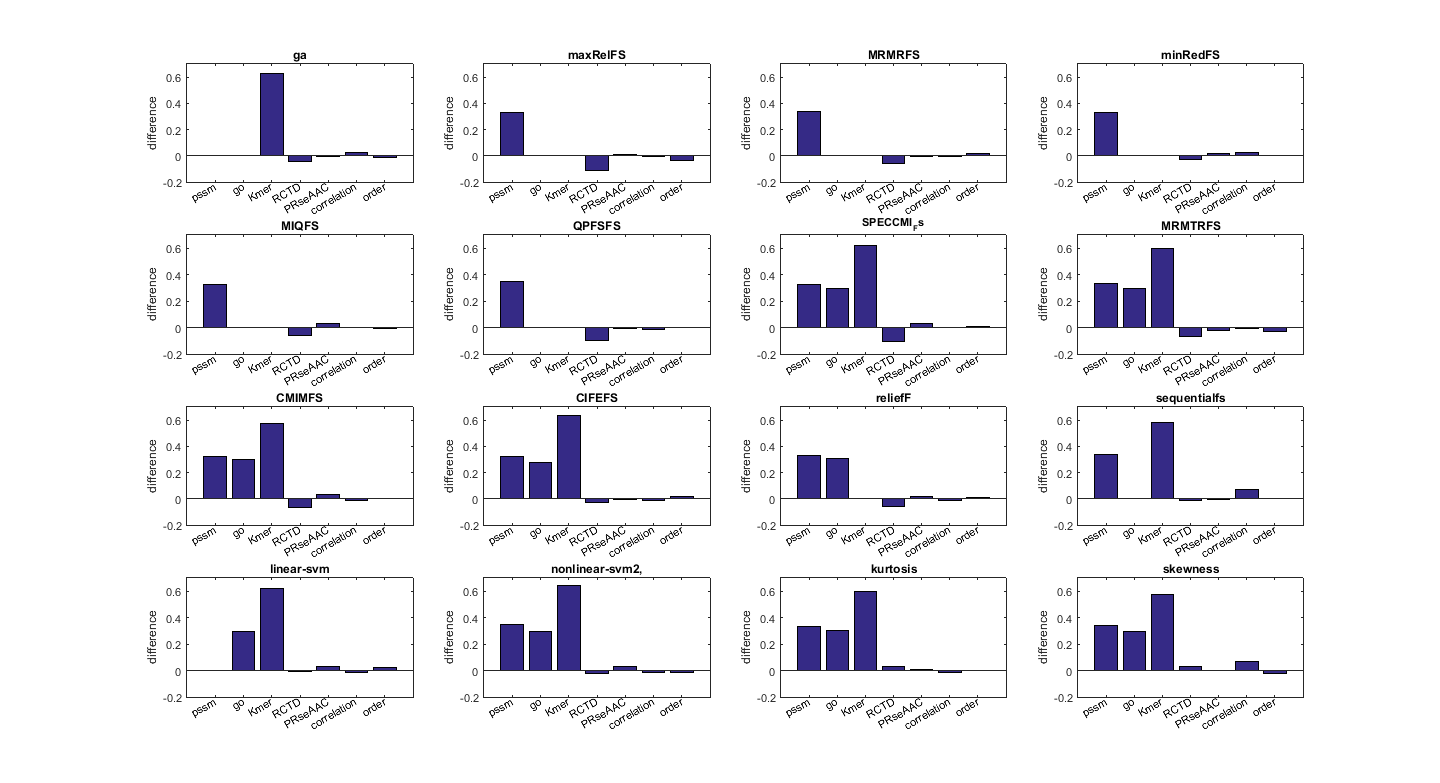


Supp Figure 6. The comparison between the accuracy of support vector machine prediction and that of single class feature prediction after selecting the top 30 features. For each graph, the selection method is arranged from left to right and from top to bottom. They are GA, and there are nine selection methods of mutual information, relief, sequentialfs, linear SVM, nonlinear SVM, kurtosis and sketchness. The horizontal axis represents sequence features, which are PSSM, go, Kmer, RCTD, PRseAAC, correlation, order and position respectively.


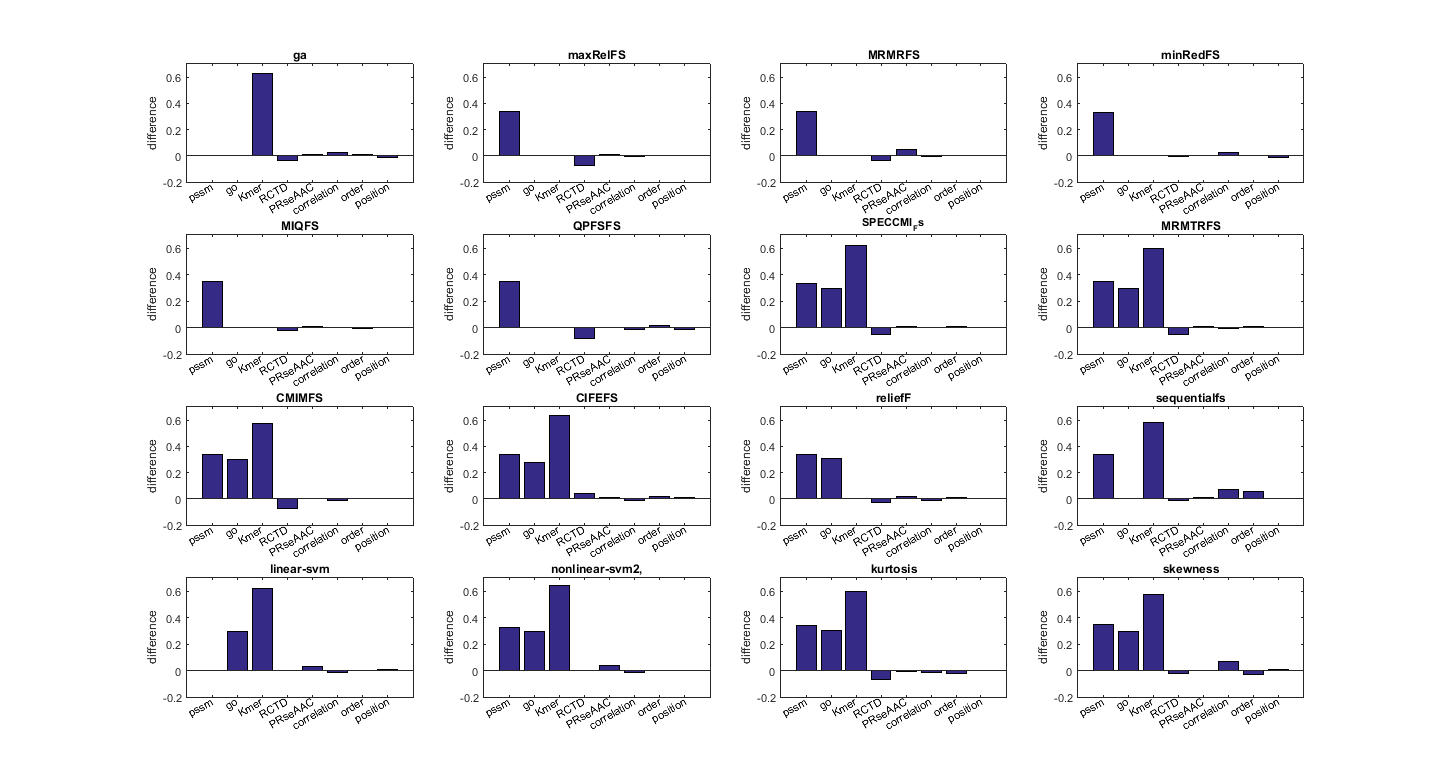


Supp Figure 7. The comparison between the accuracy of support vector machine prediction and that of single class feature prediction after selecting the top 40 features. For each graph, the selection method is arranged from left to right and from top to bottom. They are GA, and there are nine selection methods of mutual information, relief, sequentialfs, linear SVM, nonlinear SVM, kurtosis and sketchness. The horizontal axis represents sequence features, which are PSSM, go, Kmer, RCTD, PRseAAC, correlation, order and position respectively.


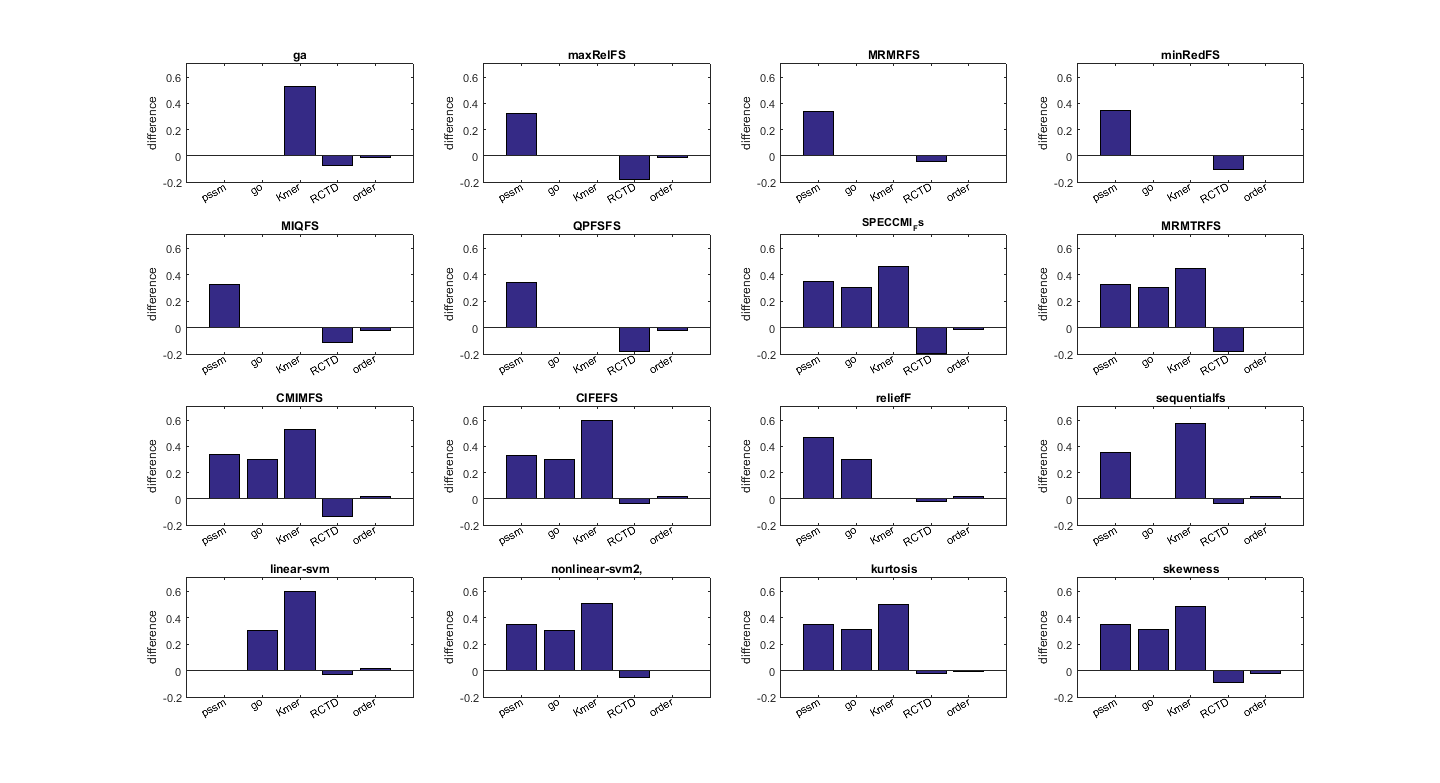


Supp Figure 8. The comparison between the accuracy of support vector machine prediction and that of single class feature prediction after selecting the top 50 features. For each graph, the selection method is arranged from left to right and from top to bottom. They are GA, and there are nine selection methods of mutual information, relief, sequentialfs, linear SVM, nonlinear SVM, kurtosis and sketchness. The horizontal axis represents sequence features, which are PSSM, go, Kmer, RCTD, PRseAAC, correlation, order and position respectively.


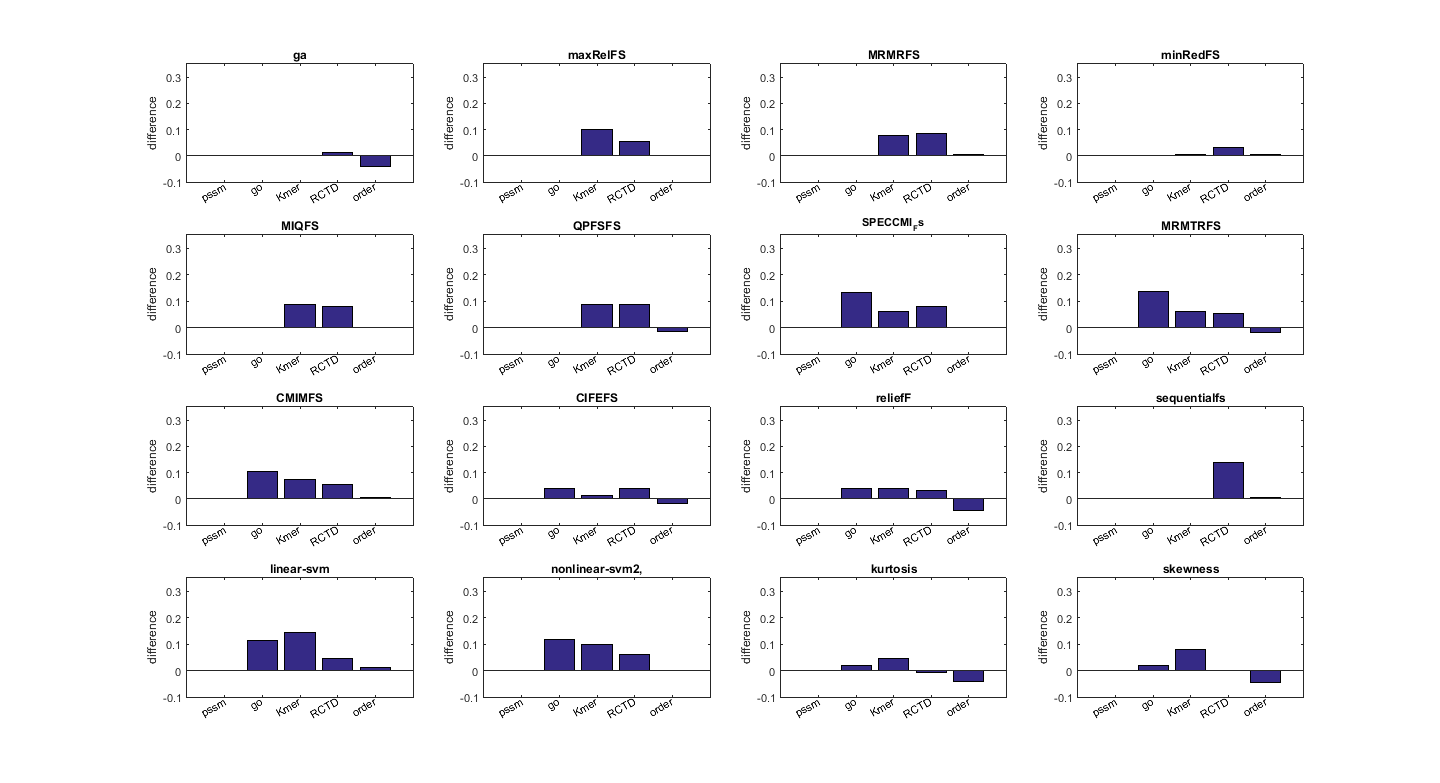


Supp Figure 9. The comparison between the accuracy of support vector machine prediction and that of single class feature prediction after selecting the top 20 features. For each graph, the selection method is arranged from left to right and from top to bottom. They are GA, and there are nine selection methods of mutual information, relief, sequentialfs, linear SVM, nonlinear SVM, kurtosis and sketchness. The horizontal axis represents sequence features, which are PSSM, go, Kmer, RCTD, PRseAAC, correlation, order and position respectively.


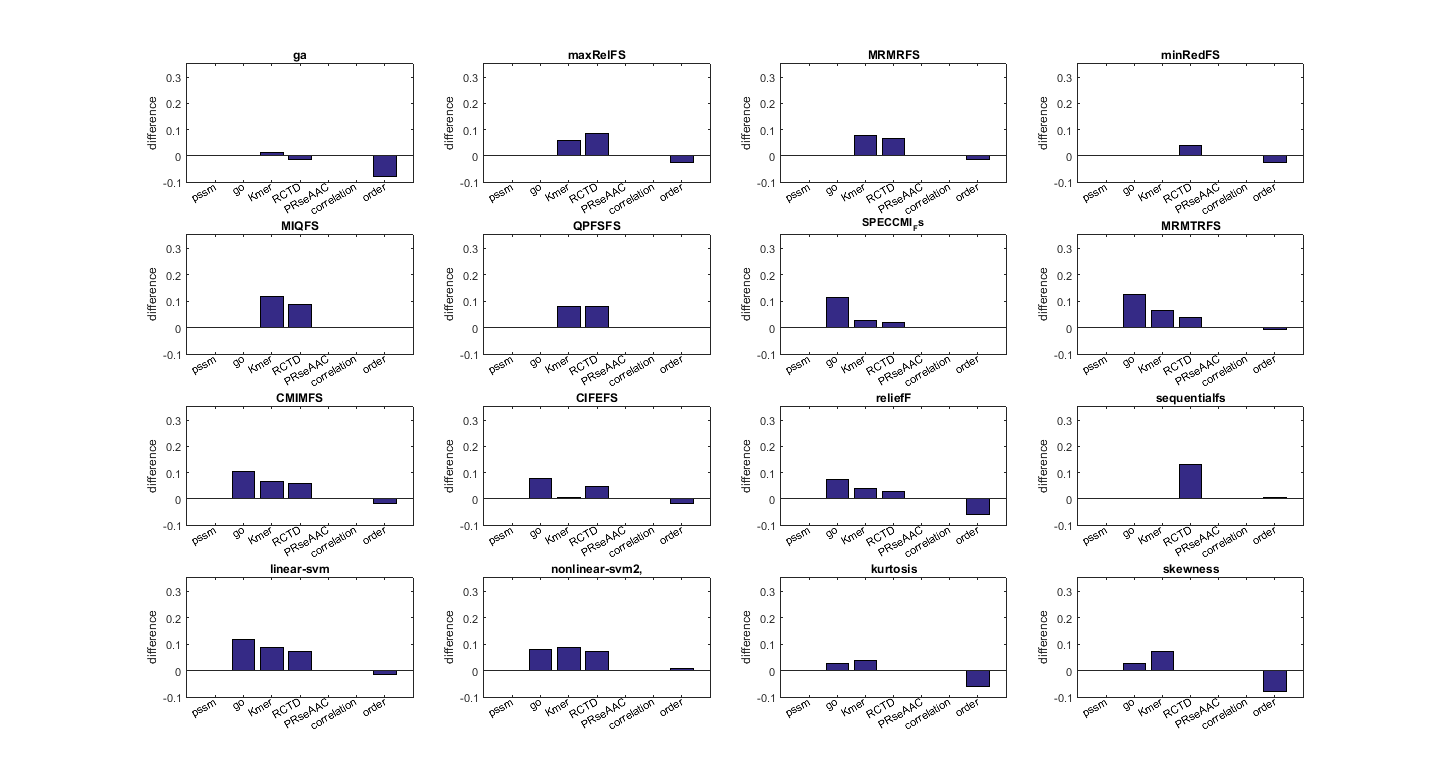


Supp Figure 10. The comparison between the accuracy of support vector machine prediction and that of single class feature prediction after selecting the top 30 features. For each graph, the selection method is arranged from left to right and from top to bottom. They are GA, and there are nine selection methods of mutual information, relief, sequentialfs, linear SVM, nonlinear SVM, kurtosis and sketchness. The horizontal axis represents sequence features, which are PSSM, go, Kmer, RCTD, PRseAAC, correlation, order and position respectively.


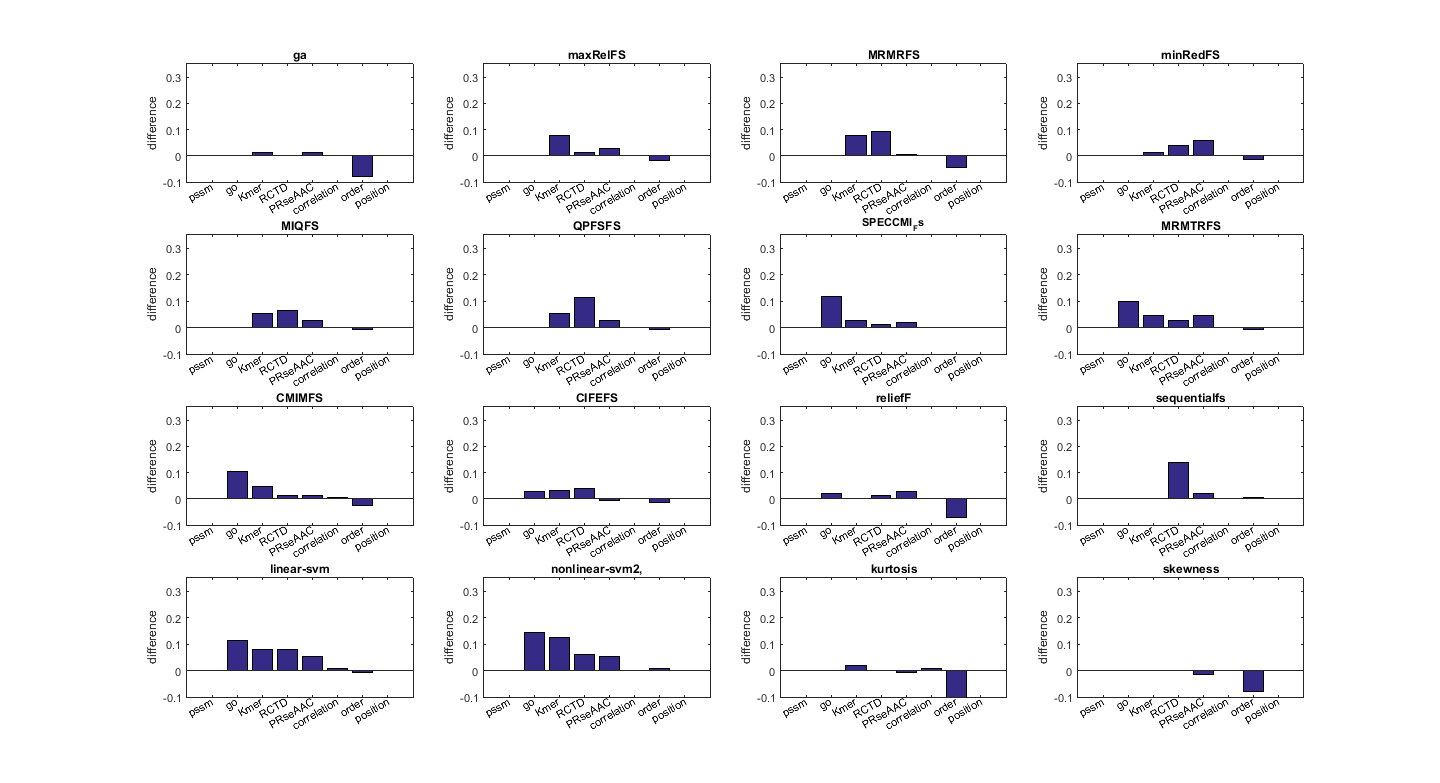


Supp Figure 11. The comparison between the accuracy of support vector machine prediction and that of single class feature prediction after selecting the top 40 features. For each graph, the selection method is arranged from left to right and from top to bottom. They are GA, and there are nine selection methods of mutual information, relief, sequentialfs, linear SVM, nonlinear SVM, kurtosis and sketchness. The horizontal axis represents sequence features, which are PSSM, go, Kmer, RCTD, PRseAAC, correlation, order and position respectively.


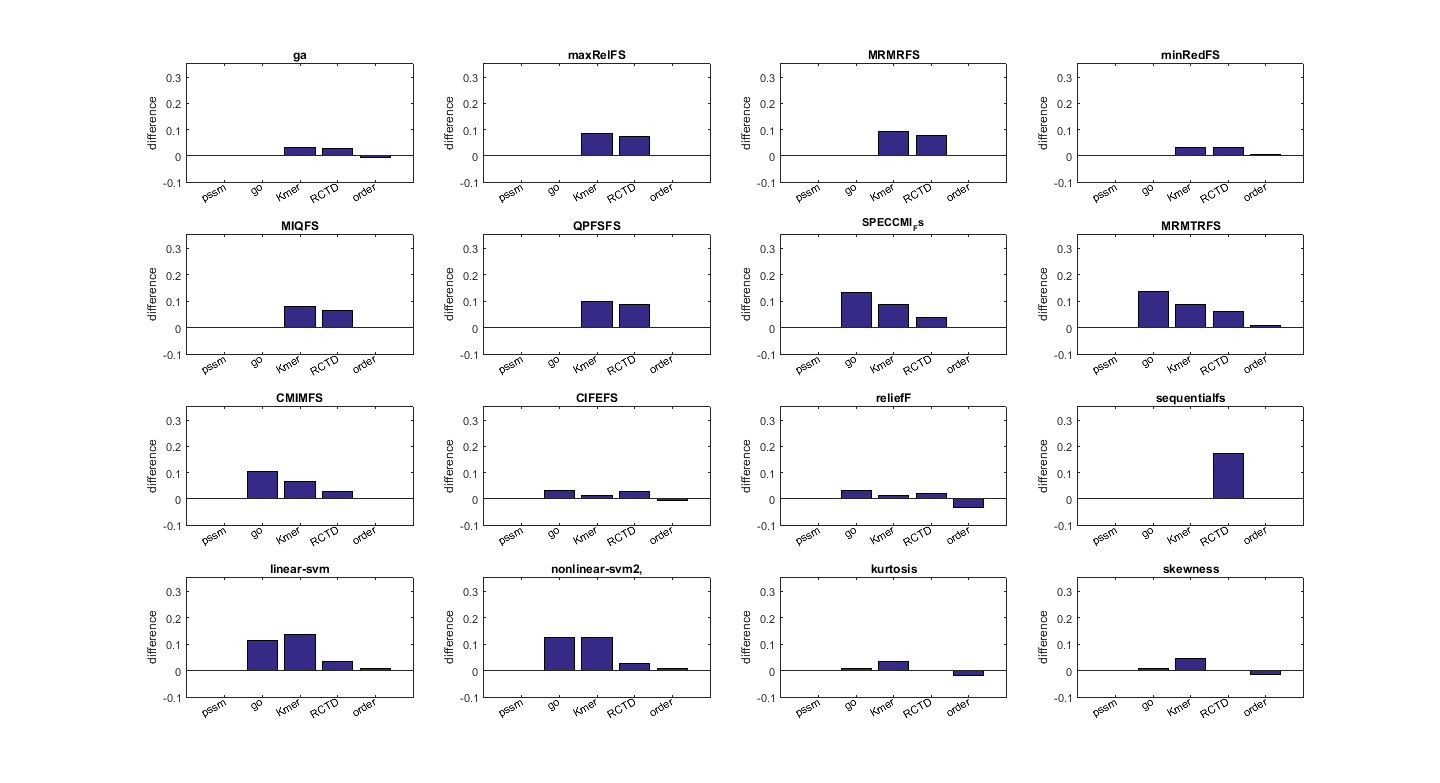


Supp Figure 12. The comparison between the accuracy of support vector machine prediction and that of single class feature prediction after selecting the top 50 features. For each graph, the selection method is arranged from left to right and from top to bottom. They are GA, and there are nine selection methods of mutual information, relief, sequentialfs, linear SVM, nonlinear SVM, kurtosis and sketchness. The horizontal axis represents sequence features, which are PSSM, go, Kmer, RCTD, PRseAAC, correlation, order and position respectively.


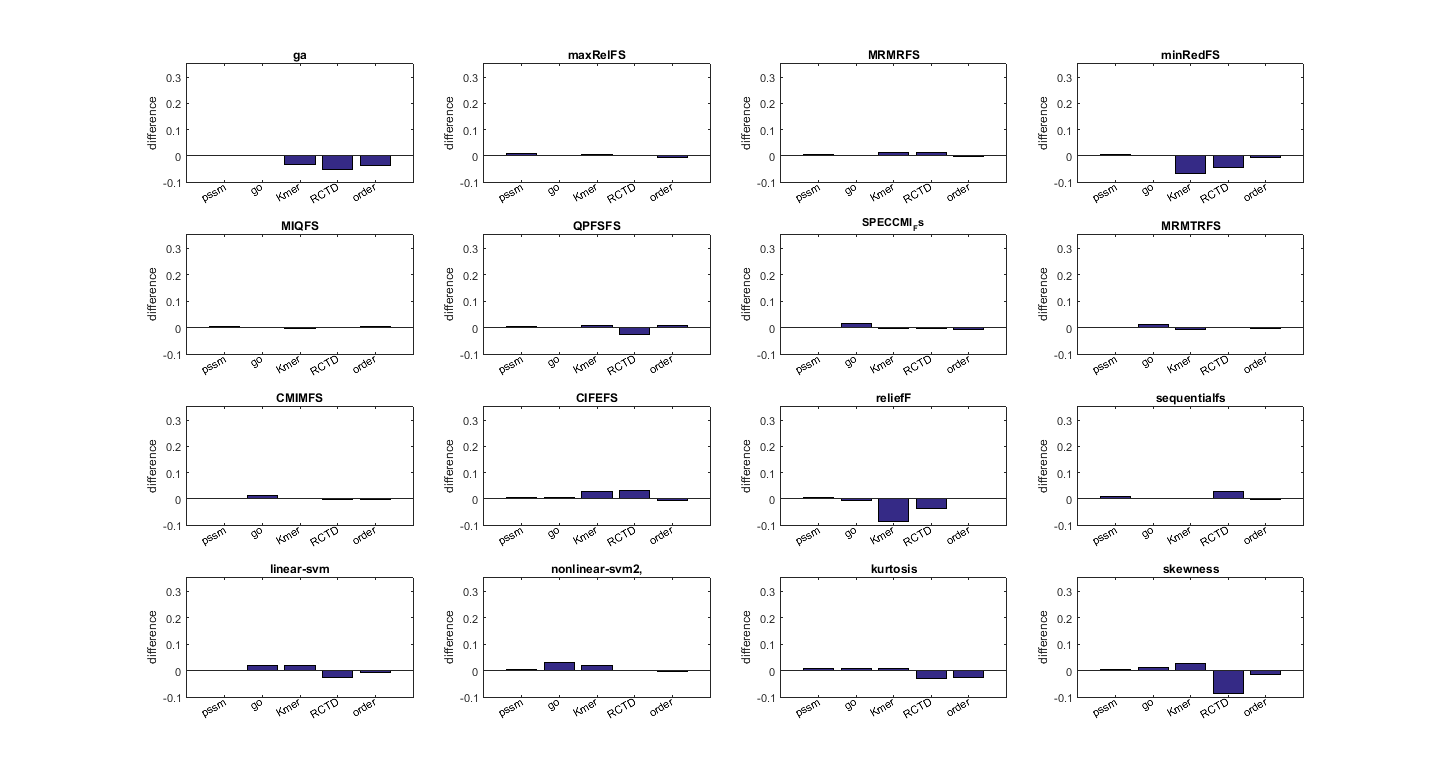


Supp Figure 13. The comparison between the accuracy of support vector machine prediction and that of single class feature prediction after selecting the top 20 features. For each graph, the selection method is arranged from left to right and from top to bottom. They are GA, and there are nine selection methods of mutual information, relief, sequentialfs, linear SVM, nonlinear SVM, kurtosis and sketchness. The horizontal axis represents sequence features, which are PSSM, go, Kmer, RCTD, PRseAAC, correlation, order and position respectively.


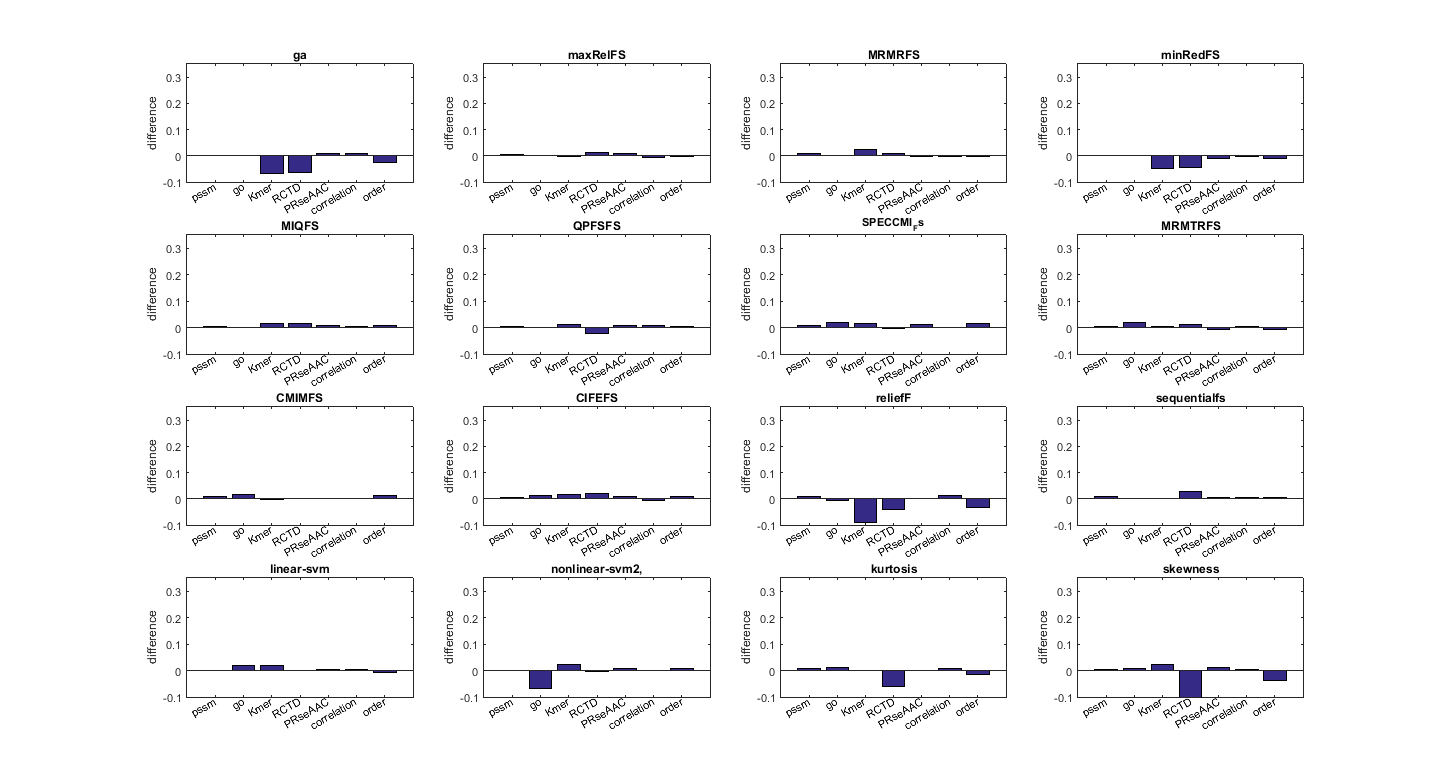


Supp Figure 14. The comparison between the accuracy of support vector machine prediction and that of single class feature prediction after selecting the top 30 features. For each graph, the selection method is arranged from left to right and from top to bottom. They are GA, and there are nine selection methods of mutual information, relief, sequentialfs, linear SVM, nonlinear SVM, kurtosis and sketchness. The horizontal axis represents sequence features, which are PSSM, go, Kmer, RCTD, PRseAAC, correlation, order and position respectively.


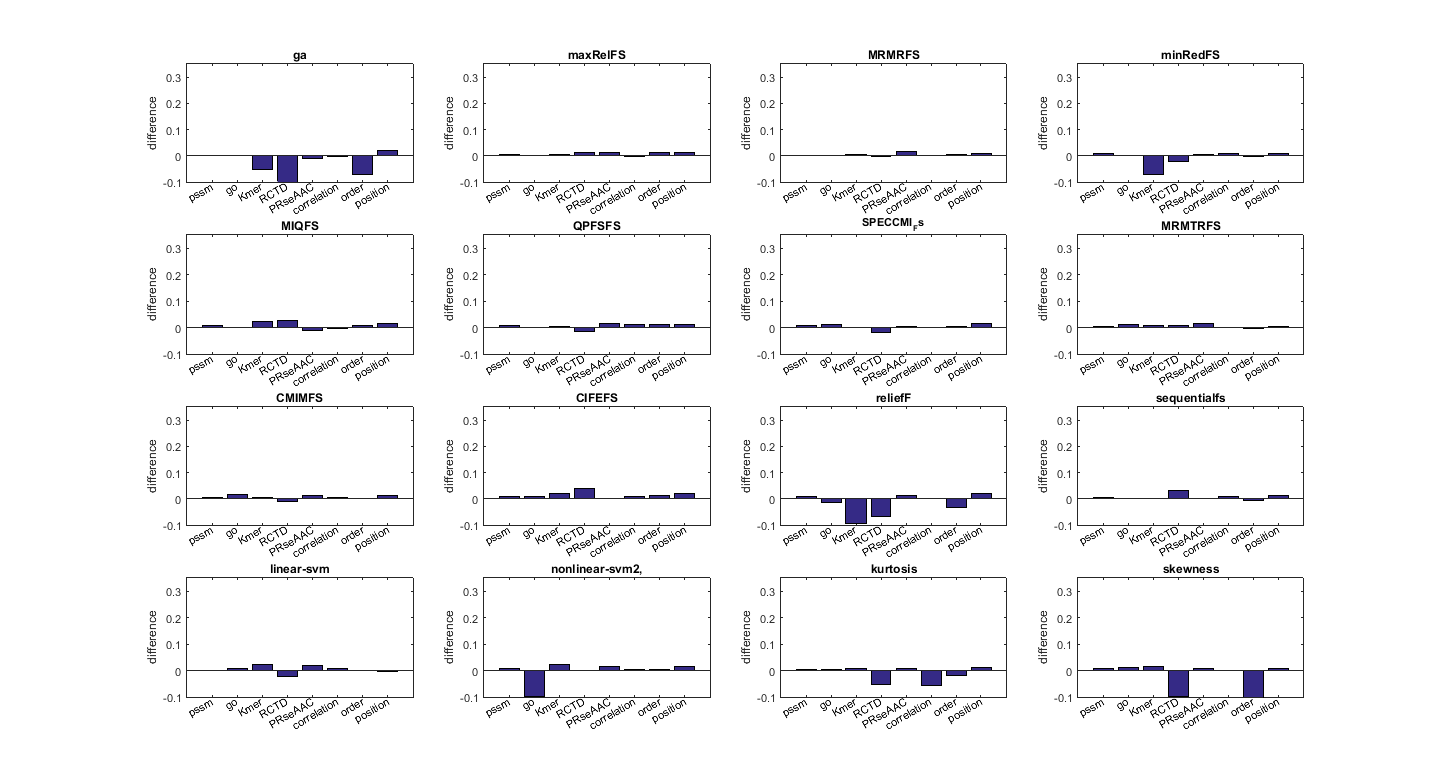


Supp Figure 15. The comparison between the accuracy of support vector machine prediction and that of single class feature prediction after selecting the top 40 features. For each graph, the selection method is arranged from left to right and from top to bottom. They are GA, and there are nine selection methods of mutual information, relief, sequentialfs, linear SVM, nonlinear SVM, kurtosis and sketchness. The horizontal axis represents sequence features, which are PSSM, go, Kmer, RCTD, PRseAAC, correlation, order and position respectively.


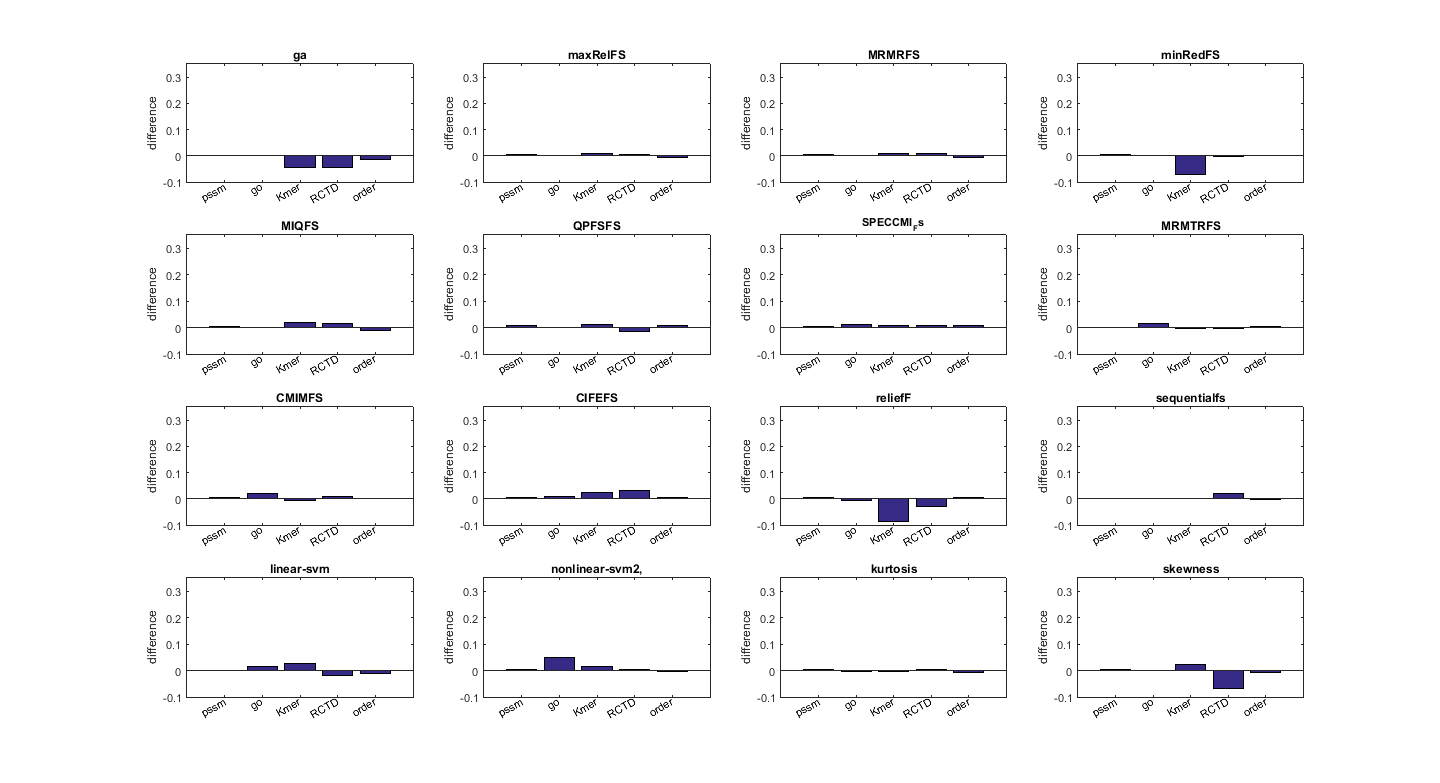


Supp Figure 16. The comparison between the accuracy of support vector machine prediction and that of single class feature prediction after selecting the top 50 features. For each graph, the selection method is arranged from left to right and from top to bottom. They are GA, and there are nine selection methods of mutual information, relief, sequentialfs, linear SVM, nonlinear SVM, kurtosis and sketchness. The horizontal axis represents sequence features, which are PSSM, go, Kmer, RCTD, PRseAAC, correlation, order and position respectively.

1. * Corresponding author. E-mail addresses: [daiailiu04@yahoo.com (Q](mailto:daiqi@zstu.edu.cn%20(Q) Dai) or [zhangweicse@zstu.edu.cn](mailto:zhangweicse@zstu.edu.cn) (W Zhang) [↑](#footnote-ref-0)
